# Determinants of self-efficacy in wrestling coaches: psychological resilience and proactive personality

**DOI:** 10.3389/fspor.2026.1770227

**Published:** 2026-04-01

**Authors:** Mustafa Serdar Terekli, Murat Yalcin Besiktas, Serda Örnek, Mert Erkan, Nalan Aksakal

**Affiliations:** 1 Faculty of Sport Sciences, Department of Physical Education Teaching, Fenerbahce University, Istanbul, Türkiye; 2 Faculty of Sport Sciences, Department of Coach Education, Fenerbahce University, Istanbul, Türkiye; 3 Faculty of Sport Sciences, Department of Sport Management, Fenerbahce University, Istanbul, Türkiye; 4 Faculty of Sport Sciences, Department of Physical Education, Eskisehir Technical University, Eskisehir, Türkiye

**Keywords:** coaching, leadership, personality, resilience, self-efficacy

## Abstract

**Background and objective:**

Coaching efficacy is a key psychological resource influencing coaches’ professional effectiveness and leadership behaviors in sports. Identifying individual characteristics that contribute to coaching efficacy is especially important in high-demand sports like wrestling. This study aimed to examine relationships between self-efficacy, psychological resilience, and proactive personality among wrestling coaches, and to determine the predictive roles of resilience and proactive personality on coaching self-efficacy.

**Methods:**

A quantitative relational survey design was employed. The sample included 116 active wrestling coaches from Eskişehir, Kütahya, Tokat, and Konya. Data were collected using the Coaching Efficacy Scale II, Psychological Resilience Scale III-R, and Short Proactive Personality Scale, all adapted into Turkish. Analyses were performed with SPSS 23.0, including Pearson correlation, multiple regression, one-way ANOVA.

**Results:**

Significant positive relationships were found between coaching self-efficacy, psychological resilience, and proactive personality. Multiple regression analysis indicated that psychological resilience (*β* = .42) and proactive personality (*β* = .38) significantly predicted coaching self-efficacy, collectively explaining 46% of the variance (R = .68, *R*^2^ = .46, *p* < .001).

**Conclusion:**

Enhancing psychological resilience and proactive personality is crucial for strengthening wrestling coaches’ self-efficacy perceptions. Improving these psychological resources can enhance professional effectiveness, leadership behaviors, and positively influence athletes. These findings offer valuable implications for coach education programs and applied sport psychology interventions.

## 1 Introduction

Achieving sustained success in sport requires not only the possession of advanced physical and technical skills but also the systematic and long-term development of these competencies within a structured and sustainable framework. Sporting success should therefore be considered not as a momentary outcome, but as the result of a planned and continuous developmental process. In this context, the role of the coach is of critical importance, as coaches are responsible for analyzing athletes’ individual characteristics, psychological needs, and developmental levels, while guiding performance in line with scientific principles (1). Coaches are not merely responsible for transferring technical and tactical knowledge; rather, they function as professional guides who structure learning environments, shape motivation, and support athletes’ psychological development (2). Feedback strategies, goal-setting behaviors, and the level of psychological support provided by coaches play a decisive role in athletes’ performance perceptions and self-efficacy levels. Previous research indicates that effective coaching behaviors are associated not only with performance outcomes but also with athletes’ sport commitment, burnout levels, and long-term participation (3). When the coach–athlete relationship is grounded in trust and psychological support, athletes demonstrate greater stress tolerance and more stable performance patterns (4). Within this framework, self-efficacy has been identified as a core psychological construct influencing performance-related behaviors. Self-efficacy refers to an individual's belief in their capability to successfully perform specific tasks and is considered a fundamental motivational resource guiding behavior (5). Individuals with high self-efficacy demonstrate greater persistence, exert higher effort when facing challenges, and employ more effective coping strategies (6). In sport psychology, self-efficacy has been widely examined as a determinant of performance consistency, goal commitment, and adaptive behavior in competitive environments (7). Recent studies have increasingly focused on identifying psychological resources that contribute to the development of self-efficacy. Among these resources, psychological resilience and proactive personality have received growing attention. Psychological resilience is defined as an individual's capacity to adapt to stress and recover from challenging life conditions (8, 9). Resilient individuals tend to interpret difficulties as opportunities for growth rather than threats, which strengthens their self-efficacy through adaptive coping and cognitive reframing processes (10). Empirical evidence from sport and organizational contexts suggests that individuals with higher levels of psychological resilience cope more effectively with stress and maintain determination in goal-directed behavior (11). In addition, proactive personality—defined as the tendency to take initiative, shape environmental conditions, and actively seek opportunities—has been identified as another important predictor of self-efficacy (9, 12). Proactive individuals do not passively respond to environmental demands; instead, they anticipate potential challenges and engage in solution-oriented behaviors. This proactive orientation enhances mastery experiences and perceived control, both of which are central sources of self-efficacy (13). Previous research has demonstrated that proactive personality is associated with higher performance levels, stronger persistence, and greater motivation across various professional domains (14, 15). Although self-efficacy, psychological resilience, and proactive personality have been examined in athletes and different occupational groups, studies focusing specifically on coaches—particularly wrestling coaches—remain limited. Wrestling is a sport characterized by high physical demands, intense competitive pressure, and strict performance expectations. Coaches in this context are required not only to manage technical and tactical processes but also to provide continuous psychological support to athletes (16). Despite these demands, limited empirical evidence exists regarding how wrestling coaches’ psychological resilience and proactive personality contribute to their self-efficacy perceptions. Understanding the interplay between these psychological variables is essential for both theoretical advancement and applied coach education. Previous studies suggest that examining self-efficacy together with psychological resources such as resilience and proactive personality provides a more comprehensive explanation of individuals’ ability to cope with stress and sustain performance over time (17, 18). However, the extent to which these variables predict self-efficacy among wrestling coaches has not been sufficiently explored. Therefore, the present study aims to examine the relationships between self-efficacy, psychological resilience, and proactive personality among wrestling coaches and to determine the predictive roles of psychological resilience and proactive personality on coaching self-efficacy. Additionally, the study investigates whether these variables differ according to demographic characteristics such as age, coaching level, and years of coaching experience.

## 2 Methodology

### 2.1 Research design

This study employed a correlational research design to examine the relationships among wrestling coaches’ self-efficacy, psychological resilience, and proactive personality characteristics. Correlational designs are used to determine the direction and strength of associations between two or more variables without manipulating them.

### 2.2 Participants

The study sample consisted of 116 wrestling coaches working in various clubs and Provincial Directorates of Youth and Sports in the provinces of Eskişehir, Kütahya, and Tokat. Participants differed in age, coaching level, and years of coaching experience. Demographic characteristics of the sample are presented in Table 1.

**Table 1:** Demographic characteristics of the research group.

Variables	Categories	*N*	%
Age	25–29	47	40.52
	30–34	34	29.31
	35–39	23	19.83
	40–44	8	6.89
	45 and above	4	3.45
	Total	**116**	**100**
Coaching level	Level 1 (Assistant Coach)	33	28.44
	Level 2 (Coach)	59	50.86
	Level 3 (Senior Coach)	13	11.21
	Level 4 (Head Coach)	7	6.03
	Level 5 (Technical Director)	4	3.45
	Total	**116**	**100**
Years of experience	1–5	42	36.21
	6–11	43	37.07
	12–17	15	12.93
	18 and above	16	13.79
Total		**116**	**100**

*N* represents the number of participants in each category. Percentages (%) indicate the proportional distribution of participants across age groups, coaching certification levels, and years of coaching experience. Coaching levels are classified according to the national coaching certification system.

### 2.3 Data collection instruments

Data were collected using a demographic information form and three standardized measurement instruments.

#### 2.3.1 Coaching efficacy scale–II

The Coaching Efficacy Scale–II, originally developed by (2) and modified by (19), was used to assess coaches’ self-efficacy perceptions. The scale consists of 18 items across five subdimensions: motivation, game strategies, technical instruction, character building, and physical conditioning. Items are rated on a 4-point Likert-type scale ranging from 1 (Low) to 4 (Complete). The modified version includes revisions to item wording and response format to enhance clarity and applicability.

#### 2.3.2 Psychological resilience scale III-R (personal views survey III-R)

Psychological resilience was measured using the Psychological Resilience Scale III-R, developed by (8) and adapted into Turkish by (20). The scale includes 18 items across three subdimensions: commitment, control, and challenge. Each subdimension consists of six items, including both positively and negatively worded statements. Reported internal consistency coefficients for the overall scale and subdimensions exceed the acceptable threshold of.70.

#### 2.3.3 Abbreviated proactive personality scale

Proactive personality characteristics were assessed using the Abbreviated Proactive Personality Scale, developed by (9) and adapted into Turkish by (21). The scale consists of 10 items rated on a 7-point Likert scale ranging from 1 (Strongly disagree) to 7 (Strongly agree), with higher scores indicating higher levels of proactive personality.

### 2.4 Procedure

Data were collected through voluntary participation. Prior to data collection, participants were informed about the purpose of the study, and informed consent was obtained. All questionnaires were administered in accordance with ethical research principles.

### 2.5 Data analysis

Statistical analyses were conducted using SPSS 23 Descriptive statistics were used to summarize demographic variables and scale scores. Pearson's product–moment correlation coefficient was employed to examine relationships among self-efficacy, psychological resilience, and proactive personality. Multiple linear regression analysis was conducted to determine the predictive effects of psychological resilience and proactive personality on self-efficacy. One-way analysis of variance (ANOVA) was used to examine differences in study variables according to age, coaching level, and years of coaching experience.

## 3 Findings

This section presents the statistical analyses conducted to address the research questions and summarizes the main findings.

### 3.1 Confirmatory factor analysis

Confirmatory factor analysis (CFA) was conducted to verify the factor structures of the Coaching Efficacy Scale–II, Psychological Resilience Scale, and Abbreviated Proactive Personality Scale. Model fit was evaluated using *χ*^2^/df, RMSEA, SRMR, CFI, TLI, and GFI indices. The CFA results indicated acceptable to good model fit for all measurement instruments (Tables 2–4) (34).

**Table 2:** Confirmatory factor analysis (CFA) Fit indices for the coaching efficacy scale II.

Fit index	Value
*χ* ^2^	320.40
df	120
*χ*^2^/df	2.67
RMSEA	0.064
SRMR	0.049
CFI	0.93
TLI	0.91
GFI	0.90

*χ*^2^, chi-square; df, degrees of freedom; RMSEA, root mean square error of approximation; SRMR, standardized root mean square residual; CFI, comparative fit index; TLI, Tucker–Lewis index; GFI, goodness-of-fit index. Values indicate acceptable model fit for the Coaching Efficacy Scale II.

**Table 3:** Confirmatory factor analysis (CFA) values for the psychological resilience scale.

*χ* ^2^	df	*χ*^2^/df	RMSEA	SRMR	CFI	TLI	GFI
198.42	85	2.33	0.058	0.043	0.94	0.91	0.88

*χ*^2^, chi-square; df, degrees of freedom; RMSEA, root mean square error of approximation; SRMR, standardized root mean square residual; CFI, comparative fit index; TLI, Tucker–Lewis index; GFI, goodness-of-fit index. The fit indices indicate acceptable model fit for the Psychological Resilience Scale.

**Table 4:** CFA results for the abbreviated proactive personality scale.

*χ* ^2^	df	*χ*^2^/df	RMSEA	SRMR	CFI	TLI	GFI
145.76	62	2.35	0.060	0.046	0.95	0.93	0.92

*χ*^2^, chi-square; df, degrees of freedom; RMSEA, root mean square error of approximation; SRMR, standardized root mean square residual; CFI, comparative fit index; TLI, Tucker–Lewis index; GFI, goodness-of-fit index. The fit indices indicate acceptable model fit for the Abbreviated Proactive Personality Scale.

### 3.2 Demographic characteristics of the participants

The demographic characteristics of the wrestling coaches are presented in Table 1. Regarding age distribution, coaches aged 25–29 constituted the largest proportion of the sample (40.51%), followed by those aged 30–34 (29.31%) and 35–39 (22.41%). Coaches aged 45 years and above represented the smallest group (3.45%). Descriptive statistics for coaches' self-efficacy, psychological resilience, and proactive personality levels are presented in Table 5.

**Table 5:** Descriptive findings regarding coaches’ self-efficacy, psychological resilience, and proactive personality levels.

Variable	*N*	Min.	Max.	Mean ($\bar{X}$)	SD
Self-efficacy	116	2.30	5.00	3.87	0.54
Psychological resilience	116	2.45	5.00	3.92	0.49
Proactive personality	116	2.10	4.95	3.78	0.58

N, sample size; Min., minimum value; Max., maximum value; $\bar{X}$, mean; SD, standard deviation. Higher scores indicate higher levels of self-efficacy, psychological resilience, and proactive personality.

In terms of coaching certification level, the majority of participants held a Level 2 coaching license (68.44%), followed by Level 1 assistant coaches (38.28%). Higher certification levels were less represented in the sample, with Level 5 technical directors accounting for 3.44% of participants.

With respect to coaching experience, coaches with 6–11 years of professional experience formed the largest group (37.08%), followed by those with 12–17 years of experience (30.17%). Coaches with 18 years or more of experience constituted the smallest proportion of the sample (13.79%). As shown in Table 6, ANOVA results indicate age-related differences in coaches' self-efficacy, psychological resilience, and proactive personality.

**Table 6:** ANOVA results for coaches’ self-efficacy, psychological resilience, and proactive personality by Age.

Variable	Source of Variance	SS	df	MS	F	*p*
Self-Efficacy	Between groups	1.109	3	.327	2.08	.05
	Within groups	59.311	340	.149		
	Total	60.113	352			
Psychological Resilience	Between groups	1.175	3	.626	2.98	.05
	Within groups	52.490	340	.213		
	Total	59.718	352			
Proactive Personality	Between groups	1.478	3	.647	.78	.05
	Within groups	241.517	340	.753		
	Total	249.467	352			

SS, sum of squares; df, degrees of freedom; MS, mean square.

*p* < .05.

A one-way ANOVA was conducted to examine differences in coaches’ self-efficacy, psychological resilience, and proactive personality across age groups. Self-efficacy showed a marginally significant difference, *F*(3, 340) = 2.08, *p* = .05. Psychological resilience differed significantly across age groups, *F*(3, 340) = 2.98, *p* < .05. No significant differences were found in proactive personality, *F*(3, 340) = 0.78, *p* > .05.

A one-way ANOVA examined differences across coaching levels. Psychological resilience differed significantly, *F*(3, 340) = 2.15, *p* < .05, while self-efficacy showed a marginally significant difference, *F*(3, 340) = 2.08, *p* = .05. No significant differences were found in proactive personality, *F*(3, 340) = 0.57, *p* > .05. The results of the one-way ANOVA examining differences in coaches' self-efficacy, psychological resilience, and proactive personality across coaching levels are presented in Table 7, *p < .05 indicates statistical significance.

**Table 7:** ANOVA results for coaches’ self-efficacy, psychological resilience, and proactive personality by coaching level.

Variable	Source of Variance	SS	df	MS	F	*p*
Self-efficacy	Between groups	1.387	3	.327	2.08	.03*
	Within groups	54.811	340	.149		
	Total	68.152	352			
Psychological resilience	Between groups	1.239	3	.557	2.15	.02*
	Within groups	47.710	340	.233		
	Total	49.207	352			
Proactive personality	Between groups	2.688	3	.647	.57	.09
	Within groups	270.389	340	.753		
	Total	273.077	352			

*p < .05 indicates statistical significance

As shown in Table 8, significant positive relationships were observed among coaches’ self-efficacy, psychological resilience, and proactive personality. Self-efficacy was strongly associated with resilience (*r* = .65, *p* < .01), moderately associated with proactive personality (*r* = .52, *p* < .01), and resilience was also moderately to strongly related to proactive personality (*r* = .58, *p* < .01). These results indicate that the three variables are closely interrelated.

**Table 8:** Relationships between coaches’ self-efficacy, psychological resilience, and proactive personality.

Variables	Mean (M)	SD	1	2	3
Self-efficacy	4.12	0.56	–		
Psychological resilience	3.98	0.60	.65**	–	
Proactive personality	4.05	0.58	.52**	.58**	–

**p < .01 indicates statistical significance.

As shown in Table 9, psychological resilience and proactive personality significantly predicted coaches’ self-efficacy [F(2,97) = 35.64, *p* < .001], explaining 42% of the variance (*R*^2^ = .42). Psychological resilience was the strongest predictor (*β* = .48, *p* < .001), followed by proactive personality (*β* = .35, *p* < .001). Both psychological resilience and proactive personality were significant predictors of self-efficacy.

**Table 9:** The predictive role of psychological resilience and proactive personality on coaches’ self-efficacy perceptions.

Variable	B	Std. Error	*β*	t	*p*	R	*R* ^2^
Constant	1.25	0.21	–	5.95	.000		
Psychological Resilience	0.45	0.08	0.48	6.72	.000		
Proactive Personality	0.31	0.07	0.35	5.12	.000	0.65	0.42

[F(2,97) = 35.64, *p* < .001].

As presented in Table 10, psychological resilience, proactive personality, and age significantly predict coaches’ self-efficacy (*p* < .001), explaining 45% of the variance (*R*^2^ = .45). Resilience and proactive personality are significant positive predictors (*p* < .001). Compared with the 25–29 age group, the 30–34 group is not significant, whereas the 35–39, 40–44, and 45+ groups show progressively stronger positive effects. Overall, self-efficacy increases with age, particularly after the mid-thirties.

**Table 10:** Multiple linear regression analysis results for predicting self-efficacy (by Age).

Dependent variable: self-efficacy	B	Std. Error	β	t	*p*	R	*R* ^2^	F	*p*
Constant	1.18	0.24	—	4.92	.000	0.67	0.45	18.42	.000
Psychological resilience	0.37	0.08	0.41	5.88	.000				
Proactive personality	0.30	0.07	0.33	4.91	.000				
Age (30–34)	0.11	0.09	0.12	1.24	.219				
Age (35–39)	0.19	0.08	0.21	2.37	.020				
Age (40–44)	0.26	0.10	0.24	2.60	.011				
Age (45 and above)	0.32	0.12	0.28	2.71	.008				

According to Table 11, the regression model is significant [F(6,109) = 21.42, *p* < .001] and explains 48% of the variance in coaches’ self-efficacy (*R*^2^ = .48). Psychological resilience and proactive personality are significant positive predictors (*p* < .001). Using Level 1 as the reference, Level 2 is not significant, whereas Levels 3, 4, and 5 show progressively stronger positive effects. Overall, self-efficacy increases systematically with higher coaching level, particularly from Level 3 onward.

**Table 11:** Multiple linear regression analysis results for predicting self-efficacy (coaching level).

Predictor	B	Std. Error	β	t	*p*	R	*R* ^2^	F	*p*
Constant	1.09	0.27	—	4.04	.000	0.69	0.48	21.42	.000
Psychological resilience	0.36	0.07	0.40	5.51	.000				
Proactive personality	0.32	0.07	0.35	4.87	.000				
Age (30–34)	0.11	0.09	0.12	1.24	.219				
Age (35–39)	0.19	0.08	0.21	2.37	.020				
Age (40–44)	0.26	0.10	0.24	2.60	.011				
Age (45 and above)	0.32	0.12	0.28	2.71	.008				
Level 2 (Coach)	0.10	0.09	0.11	1.12	.265				
Level 3 (Senior Coach)	0.18	0.08	0.19	2.25	.027				
Level 4 (Head Coach)	0.25	0.09	0.23	2.79	.006				
Level 5 (Technical Director)	0.31	0.11	0.26	2.81	.005				

Dependent Variable: Self-Efficacy.

Level 1 was used as the reference group.

The multiple regression analyses indicate that psychological resilience and proactive personality significantly predict coaches’ self-efficacy across all experience groups (*p* < .001), with explained variance ranging from.40 to.46. Across 1–5, 6–11, 12–17, and 18 + years of experience, psychological resilience consistently emerged as the strongest predictor, while proactive personality also showed a significant positive contribution in all models. These results show that psychological resilience and proactive personality significantly predict self-efficacy across all experience groups. Table 12 presents the regression results on the effects of psychological resilience and proactive personality on coaches' self-efficacy across experience groups.

**Table 12:** Multiple linear regression analysis results for predicting self-efficacy (based on years of experience).

Years of experience	Predictor	B	Std. Error	β	t	*p*	R	*R* ^2^	F	*p*
1–5 years	Constant	1.12	0.25	—	4.48	.000	0.67	0.45	18.92	.000
	Psychological resilience	0.38	0.09	0.41	4.22	.000				
	Proactive personality	0.30	0.08	0.35	3.71	.001				
6–11 years	Constant	1.15	0.23	—	5.02	.000	0.68	0.46	20.11	.000
	Psychological resilience	0.39	0.07	0.42	6.01	.000				
	Proactive personality	0.31	0.08	0.34	5.18	.000				
12–17 years	Constant	1.20	0.27	—	4.35	.000	0.65	0.42	16.44	.000
	Psychological resilience	0.40	0.10	0.43	4.00	.003				
	Proactive personality	0.28	0.09	0.30	3.11	.003				
18 years and above	Constant	1.25	0.29	—	4.31	.000	0.63	0.40	15.02	.000
	Psychological resilience	0.37	0.11	0.39	3.42	.001				
	Proactive personality	0.27	0.10	0.31	2.81	.006				

Examination of Table 13 shows that proactive personality is positively and significantly correlated with all dimensions of coaching self-efficacy (*p* < .01). The strongest relationship was found with Personality Development Efficacy (*r* = .56), followed by Teaching (*r* = .53), Management (*r* = .51), Psychological (*r* = .50), and Performance Efficacy (*r* = .47). Overall, these findings indicate that proactive personality is positively associated with all dimensions of coaching self-efficacy.

**Table 13:** Correlation analysis results between self-efficacy scale dimensions and proactive personality of wrestling coaches.

Dimensions	1	2	3	4	5	6	$\bar{x}$	SD
Performance Efficacy	—						4.29	0.46
Psychological Efficacy	.61**	—					4.24	0.48
Teaching Efficacy	.57**	.63**	—				4.36	0.44
Personality Development Efficacy	.52**	.59**	.65**	—			4.27	0.50
Management Efficacy	.56**	.54**	.61**	.63**	—		4.30	0.45
Proactive personality	.47**	.50**	.53**	.56**	.51**	—	4.15	0.52

**p < .01 indicates statistical significance.

Regression analysis results indicate that psychological resilience and proactive personality significantly predict coaches’ self-efficacy (*p* < .001). Psychological resilience (*β* = .44) is the strongest predictor, followed by proactive personality (*β* = .33). The model explains 46% of the variance in self-efficacy (*R*^2^ = .46). The regression analysis examining the predictive roles of psychological resilience and proactive personality on coaches' self-efficacy is presented in Table 14.

**Table 14:** Regression analysis of coaches’ self-efficacy, psychological resilience, and proactive personality.

Predictor	B	Std. Error	β	t	*p*	R	*R* ^2^	F	*p*
Constant	1.18	0.24	—	4.92	.000	0.68	0.46	29.87	.000
Psychological Resilience	0.41	0.09	0.44	6.13	.000				
Proactive Personality	0.29	0.08	0.33	5.02	.000				

Dependent Variable: Self-Efficacy.

## 4 Discussion

This study examined the relationships among coaching self-efficacy, psychological resilience, and proactive personality in wrestling coaches, and explored the predictive role of resilience and proactive personality on coaches’ self-efficacy perceptions. Overall, the findings provide strong empirical evidence that psychological resources play a central role in shaping coaching self-efficacy and contribute meaningfully to our understanding of psychological determinants of effective coaching practice (35). Consistent with Bandura's social cognitive theory, coaching self-efficacy was positively associated with both psychological resilience and proactive personality. Coaches who reported higher levels of resilience and proactive traits perceived themselves as more effective across multiple dimensions of coaching efficacy, including performance, psychological support, teaching, personality development, and management. These findings are in line with previous research suggesting that self-efficacy beliefs in sport professionals are closely linked to personal resources that facilitate confidence, persistence, and adaptive functioning in challenging environments (22). While much of the existing literature has focused on athletes, the present findings extend this body of work by demonstrating that similar psychological mechanisms operate in coaching populations. Psychological resilience emerged as the strongest predictor of coaching self-efficacy. This finding is consistent with prior studies indicating that resilience enables individuals to maintain confidence, motivation, and performance under pressure, uncertainty, and adversity—conditions that are inherent to competitive sport contexts (23, 24). Coaches with higher resilience may be better equipped to interpret stressful situations as manageable rather than threatening, thereby preserving their sense of competence and professional control. In this sense, resilience appears to function not only as a protective factor against stress but also as a resource that actively supports the development and maintenance of self-efficacy beliefs. Proactive personality also made a meaningful contribution to coaching self-efficacy. Coaches with proactive tendencies are more likely to anticipate challenges, take initiative, and actively shape their working environments, which may foster repeated mastery experiences—one of the most influential sources of self-efficacy according to social cognitive theory (5, 23). Previous research in organizational and leadership contexts has similarly shown that proactive individuals report higher self-efficacy, greater job engagement, and more adaptive performance outcomes (25, 26). The present findings suggest that these mechanisms are also relevant in sport coaching, where proactive behaviours may help coaches navigate organizational constraints, athlete demands, and performance pressures more effectively. Subgroup analyses indicated that coaching self-efficacy generally increased with age, coaching level, and years of experience. However, psychological resilience and proactive personality remained significant predictors across all subgroups. This finding suggests that professional experience alone is not sufficient to ensure high self-efficacy. Rather, experience appears to translate into perceived competence when supported by adaptive psychological characteristics (33). This interpretation aligns with previous evidence indicating that without adequate psychological resources, prolonged exposure to stressors may lead to stagnation or even erosion of confidence among coaches (27). Thus, psychological resilience and proactive personality may help explain why some coaches continue to develop and thrive over time, while others do not. The findings can also be meaningfully interpreted through the lens of self-determination theory. Coaches who possess higher levels of resilience and proactive personality may be better able to satisfy their basic psychological needs for autonomy, competence, and relatedness within demanding coaching environments. In such contexts, these psychological resources may facilitate adaptive coping, sustained engagement, and a stronger sense of professional agency, all of which are closely linked to self-efficacy perceptions. Conversely, frustration of these needs—particularly under conditions of chronic stress, limited organizational support, or persistent performance pressure—may undermine coaching self-efficacy and negatively influence coaching behaviours. Research has shown that basic psychological need frustration is associated with maladaptive outcomes such as emotional exhaustion, controlled motivation, reduced vitality, and less supportive interpersonal behaviours (28, 29). Recent evidence further suggests that need frustration may be linked to more controlling and less autonomy-supportive coaching behaviours in both health- and performance-related contexts (30, 31). Specifically, when coaches experience diminished feelings of autonomy or competence, they may be more likely to adopt directive or controlling interpersonal styles, potentially as a compensatory response to perceived environmental constraints or performance demands. Such behavioural patterns may, in turn, impair coach–athlete relationships and limit opportunities for athlete autonomy and psychological growth. Within this framework, psychological resilience may help coaches regulate emotional responses and maintain functional behaviour under pressure, while proactive personality may enable coaches to actively seek supportive conditions or adjust their coaching approach in ways that reduce need frustration. Together, these processes provide a plausible explanatory mechanism linking coaches’ psychological resources to self-efficacy and coaching behaviour within self-determination theory. Within this framework, psychological resilience may serve as a buffering mechanism that mitigates the negative impact of need frustration by facilitating emotional regulation and sustained engagement under pressure. Similarly, proactive personality may enable coaches to actively seek, negotiate, or create environments that better support their psychological needs, for example by initiating communication with stakeholders, pursuing professional development opportunities, or adapting their coaching strategies. Integrating self-determination theory with social cognitive perspectives therefore provides a stronger theoretical foundation for understanding how resilience and proactive personality contribute to coaching self-efficacy. From a practical standpoint, the findings underscore the importance of incorporating psychological skill development into coach education and professional development programs. Interventions targeting stress management, reflective practice, goal setting, leadership development, and resilience-building strategies may enhance coaches’ proactive behaviours and psychological resilience, thereby strengthening self-efficacy beliefs. Developing these psychological resources may not only improve coaches’ professional functioning but also promote more supportive, adaptive, and athlete-centred coaching behaviours, with potential downstream benefits for athlete well-being and performance (32). A key strength of this study lies in its integrated examination of coaching self-efficacy alongside psychological resilience and proactive personality, offering a more comprehensive understanding of psychological determinants of coaching effectiveness. The inclusion of coaches across different ages, experience levels, and coaching tiers also allows for a nuanced interpretation of how these variables operate across professional stages. Nevertheless, several limitations should be acknowledged. The cross-sectional design precludes causal inference, and longitudinal or experimental research is needed to examine how changes in resilience and proactive personality influence coaching self-efficacy over time. The sample was limited to wrestling coaches from selected regions, which may restrict the generalizability of the findings to other sports or cultural contexts. In addition, the exclusive use of self-report measures may introduce response biases; future studies could benefit from incorporating observational data, athlete perceptions, or qualitative approaches. Future research should examine longitudinal pathways linking psychological resources, basic psychological need satisfaction and frustration, and coaching behaviours to clarify underlying mechanisms. Expanding this line of inquiry across different sports, competitive levels, and cultural settings would enhance external validity. Moreover, intervention-based studies evaluating the effectiveness of resilience- and proactivity-focused training programs for coaches would provide valuable applied evidence for coach education and professional development initiatives.

## 5 Conclusions

The present study examined the extent to which psychological resilience and proactive personality predict self-efficacy among wrestling coaches. The findings indicate that both psychological resilience and proactive personality are significant predictors of coaching self-efficacy, with psychological resilience emerging as the strongest contributor. These results suggest that coaches’ perceptions of professional competence are shaped not only by experience and technical knowledge but also by key psychological resources.

In addition, the confirmatory factor analysis results confirmed that the Coaching Efficacy Scale II, Psychological Resilience Scale, and Proactive Personality Scale demonstrated acceptable validity and reliability within the study sample. This supports the appropriateness of the measurement instruments used to assess the targeted psychological constructs.

Overall, the findings highlight the importance of considering psychological characteristics in coach development processes. Coaching education programs may benefit from integrating practices that enhance psychological resilience and encourage proactive behavioral tendencies alongside traditional technical and tactical training. Future research should replicate these findings using larger and more diverse samples across different sports and competitive contexts to further strengthen the generalizability of the results.

## Data Availability

The original contributions presented in the study are included in the article/Supplementary Material, further inquiries can be directed to the corresponding author.
